# Supplementary material for: A seq2seq model to forecast the COVID-19 cases, deaths and reproductive R numbers in US counties
Source: medRxiv. 2021 Apr 20:2021.04.14.21255507. Preprint. [Version 1] doi: 10.1101/2021.04.14.21255507 (PMC8077584; doi:10.1101/2021.04.14.21255507)
Supplement: 1 [file NIHPP2021.04.14.21255507-supplement-1.pdf]

medRxiv preprint doi: https://doi.org/10.1101/2021.04.14.21255507; this version posted April 20, 2021. The copyright holder for this preprint (which was not certified by peer review) is the author/funder, who has granted medRxiv a license to display the preprint in perpetuity.
It is made available under a CC-BY 4.0 International license .

Supplementary Figure 1. ML setup.

A. Model selection, tuning and testing process

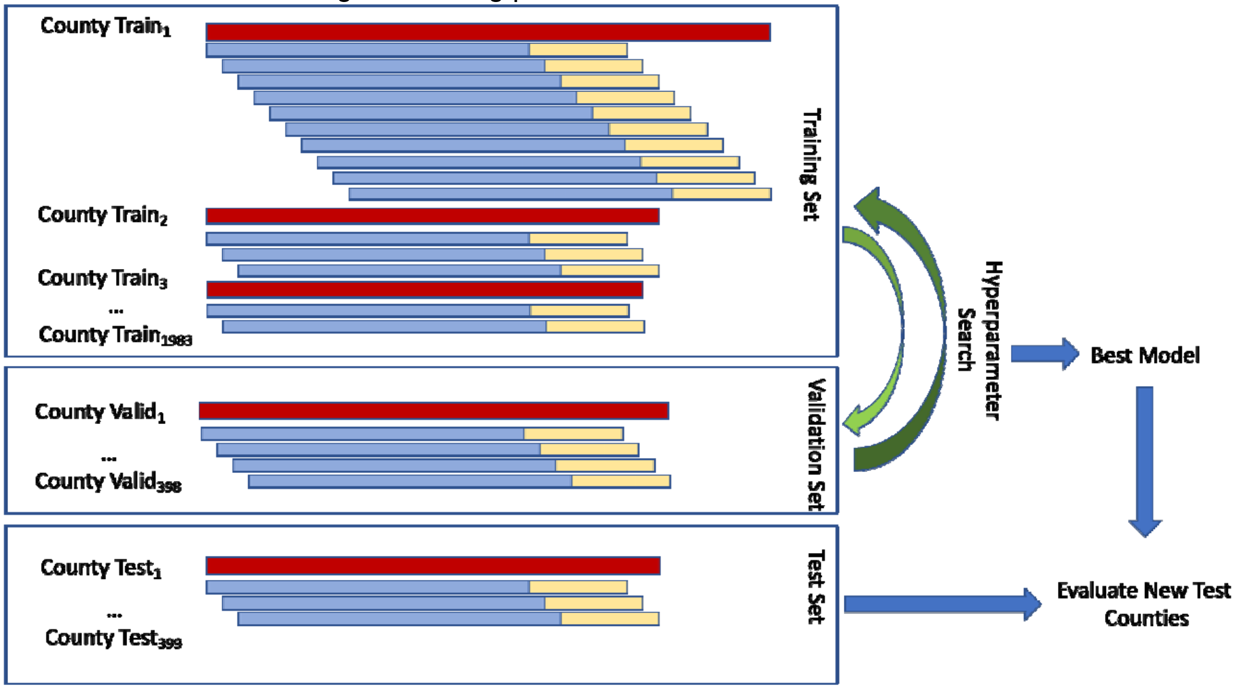

B. Making Forecast.

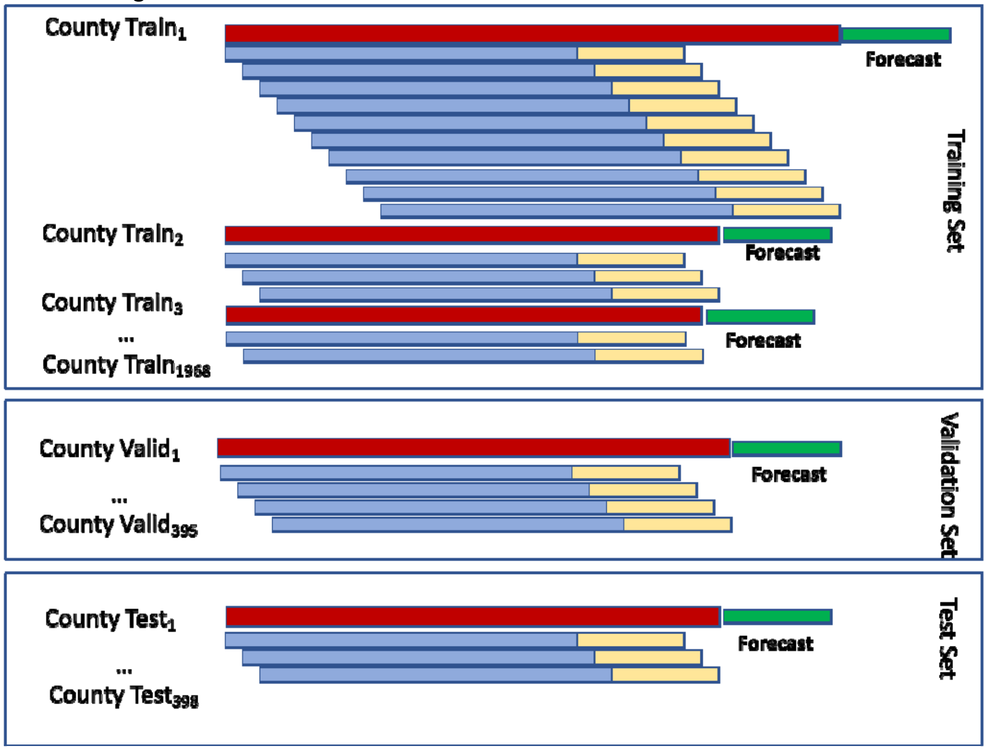

medRxiv preprint doi: https://doi.org/10.1101/2021.04.14.21255507; this version posted April 20, 2021. The copyright holder for this preprint (which was not certified by peer review) is the author/funder, who has granted medRxiv a license to display the preprint in perpetuity.
It is made available under a CC-BY 4.0 International license .

Supplementary Figure 2. HyperOpt results of various input lenghth.

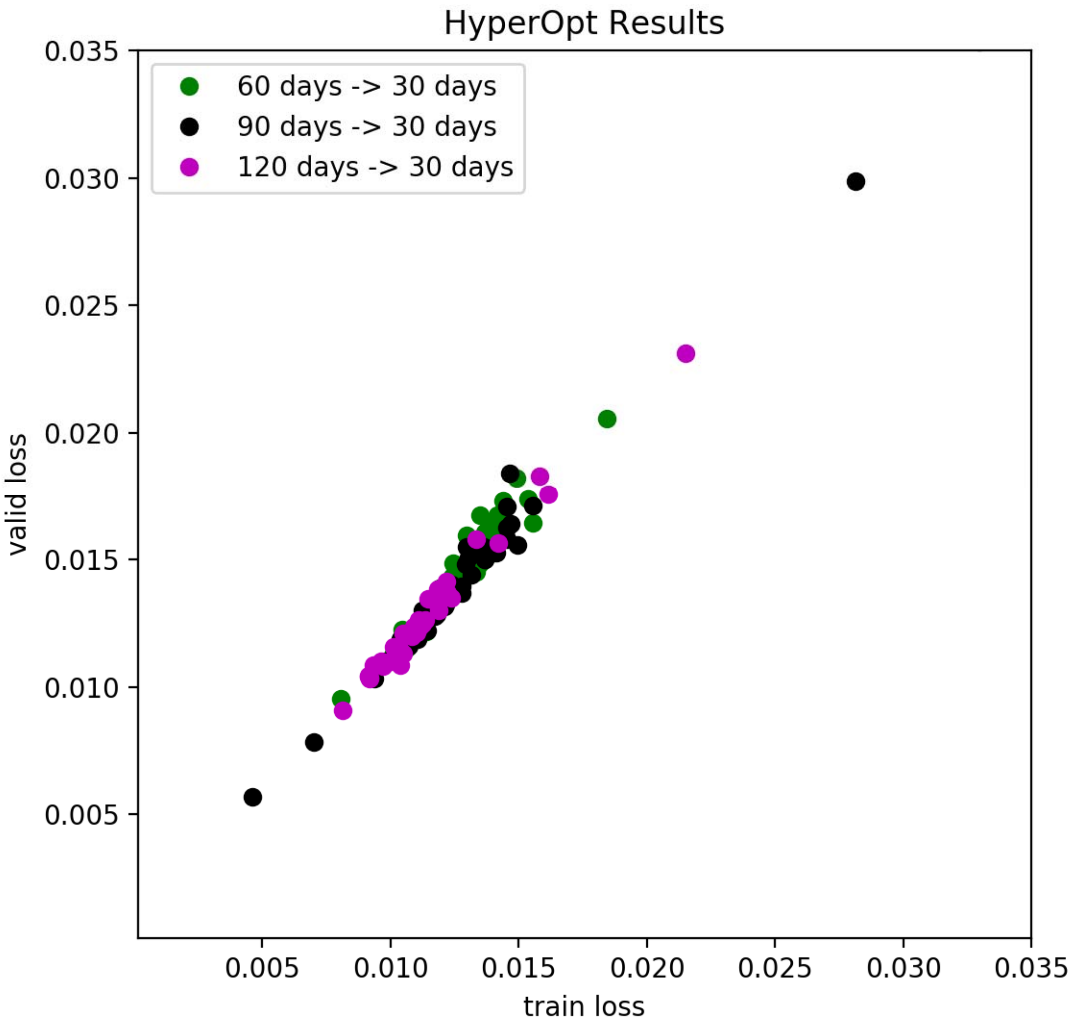

medRxiv preprint doi: https://doi.org/10.1101/2021.04.14.21255507; this version posted April 20, 2021. The copyright holder for this preprint (which was not certified by peer review) is the author/funder, who has granted medRxiv a license to display the preprint in perpetuity.
It is made available under a CC-BY 4.0 International license .

Supplementary Figure 3. **A.** Daily predicted cases (y axis) vs actual cases (x axis) over the next 30 days from the forecast date Nov 30th, 2020. **B.** Daily predicted deaths (y axis) vs actual deaths (x axis) over the 30 days forecast. **C.** Daily predicted R values (y axis) vs R calculated from the actual cases (x axis) over the 30 days forecast. **D.** Relative Error distributions.

A.

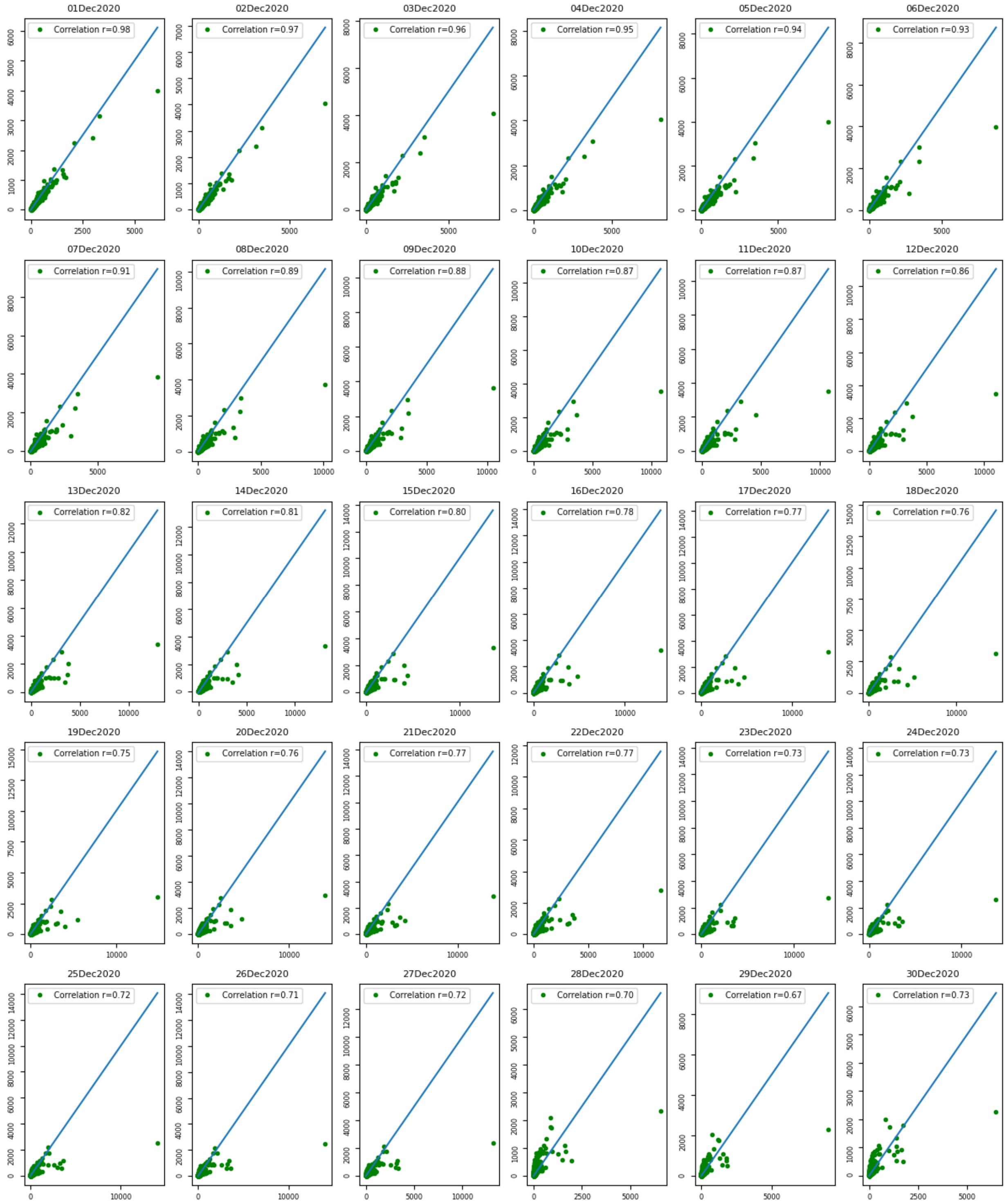

medRxiv preprint doi: https://doi.org/10.1101/2021.04.14.21255507; this version posted April 20, 2021. The copyright holder for this preprint (which was not certified by peer review) is the author/funder, who has granted medRxiv a license to display the preprint in perpetuity.
It is made available under a CC-BY 4.0 International license .

B.

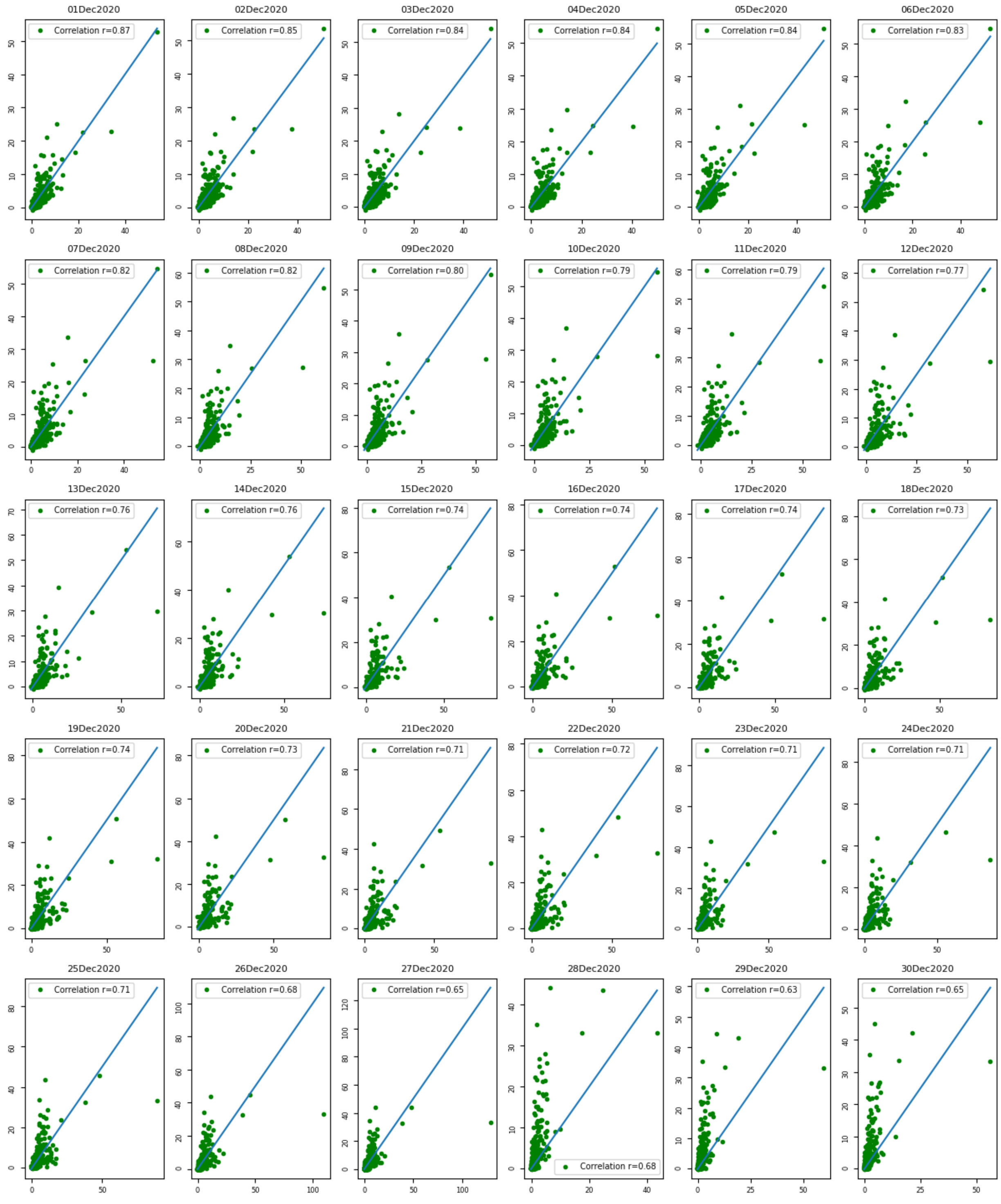

medRxiv preprint doi: https://doi.org/10.1101/2021.04.14.21255507; this version posted April 20, 2021. The copyright holder for this preprint
(which was not certified by peer review) is the author/funder, who has granted medRxiv a license to display the preprint in perpetuity.
It is made available under a CC-BY 4.0 International license .

C.

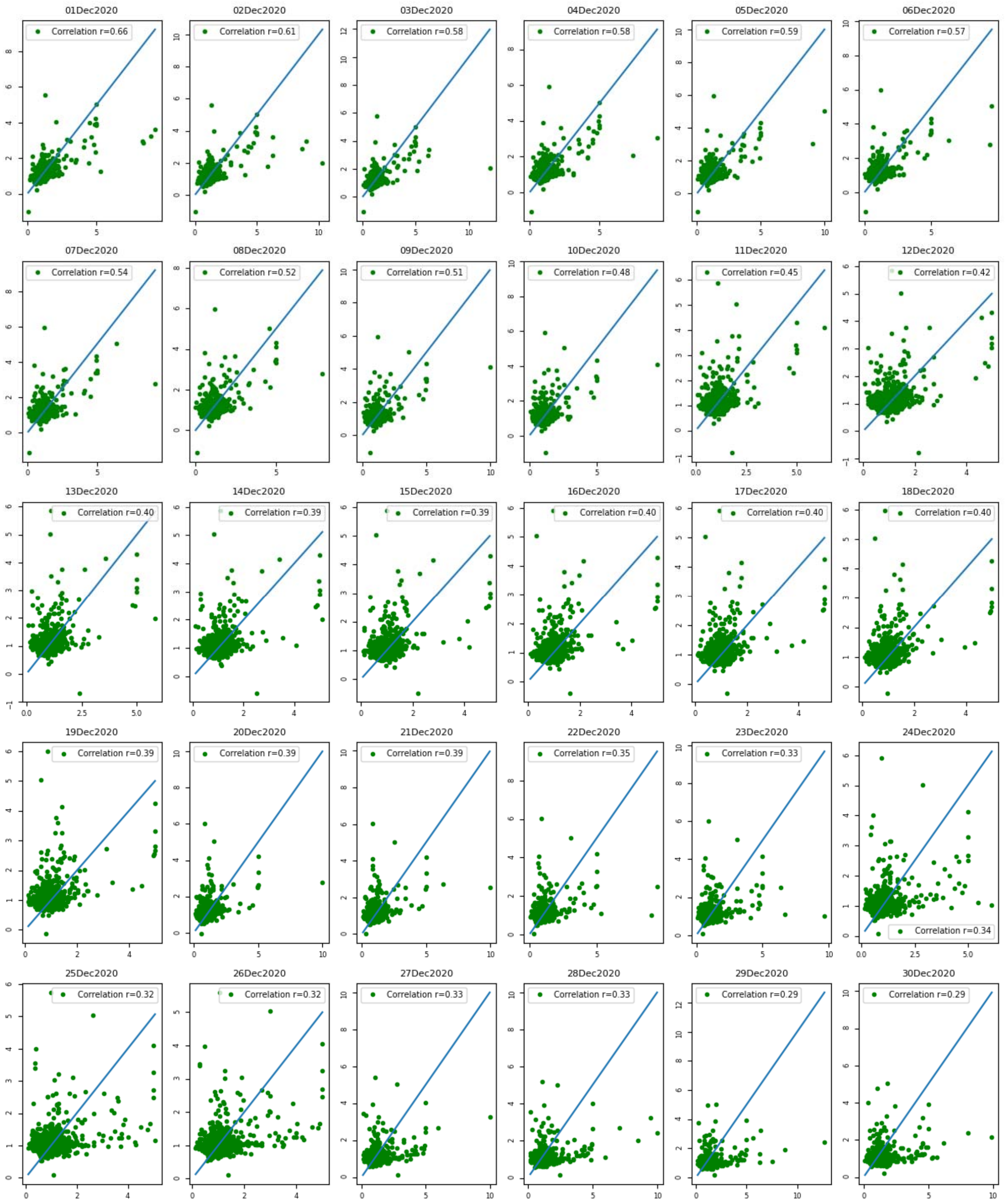

medRxiv preprint doi: https://doi.org/10.1101/2021.04.14.21255507; this version posted April 20, 2021. The copyright holder for this preprint (which was not certified by peer review) is the author/funder, who has granted medRxiv a license to display the preprint in perpetuity.
It is made available under a CC-BY 4.0 International license .

D.

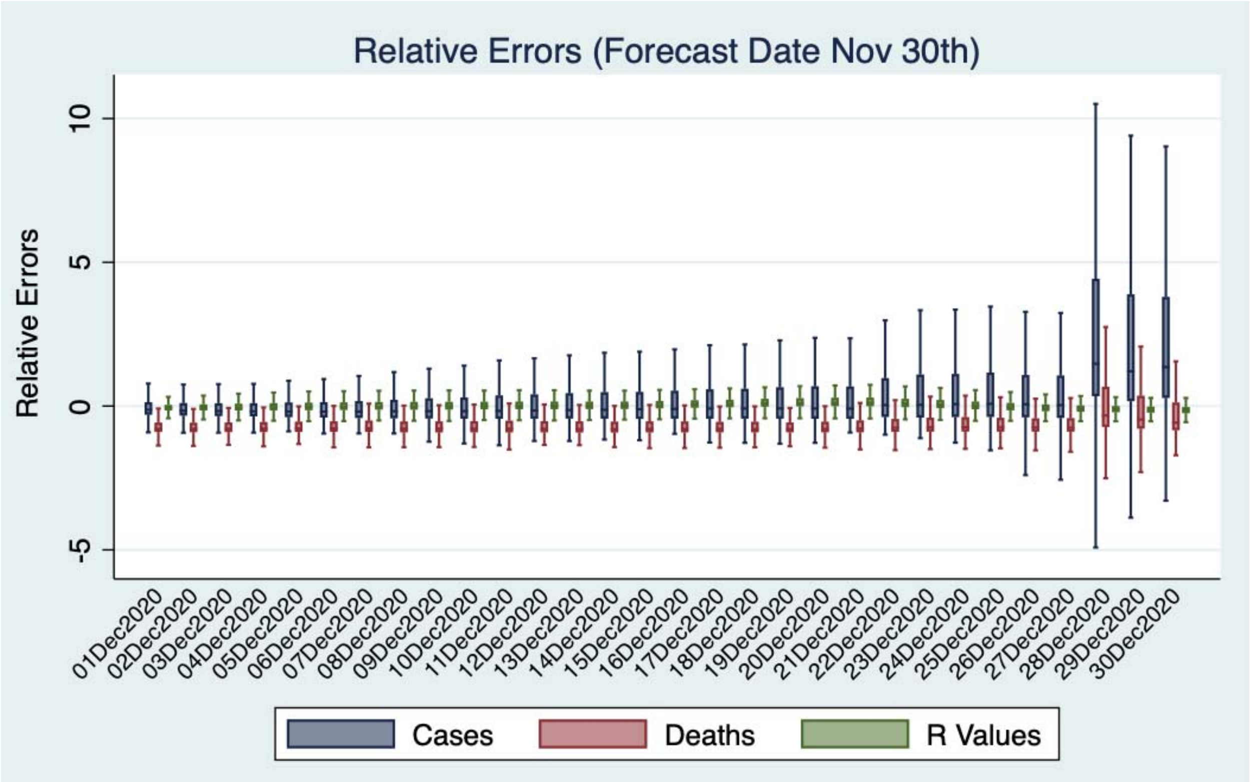

Supplementary Table 1. Median relative errors for county-level case forecast from different teams.

	Forecast Date Nov 23, 2020					Forecast Date Nov 30, 2020				
	Numbers of Counties Predicted	Median REs*				Numbers of Counties Predicted	Median REs			
Team Name		Week 1	Week 2	Week 3	Week 4		Week 1	Week 2	Week 3	Week 4
CMU	188	17.8%	19.4%	35.4%	37.5%	188	-6.3%	2.5%	25.1%	51.5%
Columbia	2743	-1.7%	-15.2%	-21.4%	-22.9%	2767	-17.1%	-26.5%	-25.1%	-12.6%
Ensemble	2769	2.2%	-12.7%	-19.0%	-15.2%	2769	-18.1%	-24.4%	-21.0%	-4.9%
ISU	2769	16.7%	-2.1%	-10.5%	-7.5%	2769	0.0%	-6.7%	-4.2%	13.3%
JHU_APL	2769	1.0%	-5.9%	-5.9%	6.4%	2769	-18.9%	-25.2%	-27.4%	-14.8%
JHU_IDD	2769	-37.9%	-45.0%	-46.2%	-47.3%	2769	-49.3%	-50.0%	-47.3%	-37.0%
LANL	2768	6.2%	-0.6%	-5.3%	-7.8%	2768	-22.3%	-26.0%	-21.5%	-8.5%
LNQ	2769	5.3%	-5.5%	-4.2%	4.4%	2769	-25.0%	-30.3%	-22.7%	-3.7%
UCLA	1233	-4.7%	-41.6%	-59.9%	-67.0%	865	-15.8%	-27.3%	-26.4%	-20.0%
UGA_CEID	2769	6.3%	-11.2%	-18.3%	-14.7%	2769	-15.7%	-21.8%	-17.9%	-2.4%
UMass_MB	457	10.4%	2.9%	1.0%	18.7%	457	-20.2%	-31.6%	-27.3%	-21.0%
UVA	2767	7.3%	-10.2%	-13.6%	-9.9%	2767	0.0%	-6.3%	-4.9%	10.2%
UpstateSU	2769	-8.9%	-20.7%	-21.2%	-10.5%	2769	-11.7%	-14.0%	-10.6%	4.1%

*Relative Errors (REs) = (predicted – observed)/observed values.
*This table included all counties that were reported by each team/model.

medRxiv preprint doi: https://doi.org/10.1101/2021.04.14.21255507; this version posted April 20, 2021. The copyright holder for this preprint (which was not certified by peer review) is the author/funder, who has granted medRxiv a license to display the preprint in perpetuity.
It is made available under a CC-BY 4.0 International license .
